# Polycystic ovary syndrome: quantifying the psychosocial burden and the predictive role of sexual trauma history in clinical outcomes

**DOI:** 10.3389/fendo.2026.1858238

**Published:** 2026-08-24

**Authors:** Victor Manickam, Vedika Pillai

**Affiliations:** Victor Manickam Foundation, Mumbai, India

**Keywords:** polycystic ovarian syndrome (PCOS), polycystic ovarian disease (PCOD), sexual trauma, psychological distress, negative self-perception, trauma-informed care, women's health, biopsychosocial model

## Abstract

Polycystic ovary syndrome (PCOS) is a complex endocrine disorder that needs to be addressed through holistic management of metabolic, hormonal, and lifestyle factors. This research study, a cross-sectional examination, reports on clinical and psychological aspects of PCOS and PCOD in a group of 75 educated women analyzed with descriptive statistics, non-parametric tests, and binary logistic regressions. To carry out this analysis, we integrated the symptom severity index (SSI) and a binary measure of past sexual trauma history. The study observed irregular menstruation in 85.3% and weight gain in 84.0% of affected participants, representing a high prevalence of these core physical symptoms. The diagnostic status shows a psychological decline: post-diagnostic changes including feeling undesirable and being more introverted (p<0.001). Sexual molestation or abuse was reported by 72% of participants. Binary logistic regression showed that higher SSI scores (Odds Ratio = 1.72; 95% Confidence Interval [CI]: [1.28,2.30]; p<0.001) and a positive history of sexual trauma (OR = 3.58; 95% CI: [1.55,8.26]; p=0.003) were independent predictors of negative self-perception at diagnosis. The study argues that biomedical interventions are not enough and there needs to be a shift to integrated trauma-informed models of care. Human Design Education and Development can provide holistic support that promotes emotional inclusion by applying the principle of Listen and Share. In addition to reproductive and metabolic characteristics, PCOS/PCOD has a major psychological impact, which is clearly observed in this research of 75 educated women. There is a high symptom intensity and immediate decline in self-concept at diagnosis, which can result in negative body image and individuals feeling compelled to isolate themselves from society as a defense mechanism. This shows statistical significance (p=0.003). The number of people with higher symptom intensity and decline in self-concept due to negative body image and defensive social connection was recorded (p<0.001). Additionally, 72% of individuals self-reported sexual trauma, indicating a critical vulnerability that independently increased the likelihood of poor self-perception, irrespective of the intensity of physical symptoms (OR = 3.58, p=0.003).This study results in the immediate need for integrated trauma-informed multidisciplinary care paradigms in PCOS management and assesses the limitations of emphasizing interventions in biomedical management.

## 1 Introduction

Two endocrine disorders that are commonly observed in women of reproductive age are polycystic ovary syndrome (PCOS) and the related condition polycystic ovarian disease (PCOD). Estimated global prevalence varies from 6% to over 20% depending on the diagnostic model and the population observed (1–3). Based on Sasikala et al. (4) and Mohmed et al. (5), prevalence was found to be about 20% in teenagers and young adults in urban India; this appears to coincide with reproductive and psychosocial phases of life.

Hyperandrogenism, ovulatory dysfunction, and polycystic ovarian morphology are the clinical hallmarks of PCOS; they are often accompanied by insulin resistance, obesity, and dyslipidemia (6, 7). There is growing evidence that PCOS is a chronic, life-altering disorder with significant psychosocial implications, even though clinical guidelines mostly focus on metabolic and reproductive care (8, 9).

Due in large part to visible symptoms like weight increase, hirsutism, acne, and infertility-related stigma, women with PCOS frequently report lower quality of life, body dissatisfaction, anxiety, depressive symptoms, and social withdrawal (8, 10). Despite this, the majority of standard care is still pharmacological and biological, frequently ignoring psychological discomfort and pre-existing vulnerabilities that affect the course of the disease and its results (1, 11).

The role of past sexual trauma is an understudied aspect of PCOS research. Chronic stress-related endocrine disruption, altered body perception, and long-term psychological dysregulation are all linked to sexual abuse (12). Few studies, especially in sociocultural contexts where disclosure is restricted, have quantitatively investigated how trauma history interacts with PCOS symptom intensity to shape psychosocial outcomes.

In order to close this gap, the current study looks at the link between the severity of somatic symptoms, psychosocial distress, and history of sexual trauma in women who self-report having PCOS or PCOD. In order to support trauma-informed, integrative care models that are in line with human-centered and holistic frameworks, the study uses composite indices and multivariate analysis to identify predictors of negative self-perception after diagnosis (13).

## 2 Literature review: pathophysiology and psychosocial determinants of PCOS and PCOD

### 2.1 Clinical distinction and hormonal pathophysiology

Despite their frequent interchangeability, PCOS and PCOD are distinguished in the literature as PCOS being a systemic endocrine-metabolic condition with long-term health consequences and PCOD a milder form of ovarian dysfunction (14, 15). Insulin resistance and compensatory hyperinsulinemia promote excessive ovarian androgen production and interfere with follicular maturation causing symptoms such as hirsutism, acne, alopecia, weight gain, and irregular menstruation, and are key contributors to the pathophysiology of PCOS (2, 7, 16, 17).

### 2.2 Regional prevalence and age-specific incidence in India

Cross-sectional studies show that PCOD and PCOS seem to be more common in India, mainly in young, educated, and urban women. Estimates from a hospital study show that up to 30% of women attending gynaecological outpatient departments were found to have critical symptoms of the conditions (4, 18). Women aged between 21 and 25 have a high incidence rate. Identity construction and increased sociocultural expectations make the psychological effects of diagnosis worse. (5, 9).

### 2.3 Systemic health risks and long-term complications

Metabolic syndrome in PCOS shows an increased lifetime risk of type 2 diabetes mellitus, hypertension, and cardiovascular disease with metabolic dysfunction, and chronic anovulation leads to a higher risk of endometrial hyperplasia and carcinoma (1, 19, 20). Various studies support the idea that PCOS is a chronic systemic condition that needs care throughout life in the current scenario.

### 2.4 Psychoneuroimmunology: the theoretical basis for holistic assessment

A theoretical framework that connects psychological stress to immunological activation and endocrine imbalance is provided by psychoneuroimmunology. According to research, long-term stress might worsen insulin resistance and hormonal disruption in PCOS by dysregulating the hypothalamic-pituitary-adrenal axis and promoting inflammatory pathways (10, 12). This reciprocal relationship implies that psychological discomfort is both a result of and a possible cause of the severity of the illness.

### 2.5 Psychological morbidity and quality of life

Reduced quality of life, a high frequency of anxiety and depressive symptoms, and low self-esteem are all frequently reported by women with PCOS; some Indian studies have shown anxiety rates as high as 90% (8, 9, 21). Infertility, hirsutism, and elevated body mass index (BMI) have been found to be important indicators of psychosocial distress because they are socially noticeable symptoms that draw stigma and encourage social disengagement.

### 2.6 Limitations of treatment and primacy of lifestyle intervention

Although pharmacological management—especially the use of oral contraceptive pills—remains a key component of PCOS treatment, it is often linked to side effects such as weight gain, mood swings, and poor long-term (1, 22). In order to enhance insulin sensitivity and restore ovulatory function, modern therapeutic guidelines place a strong emphasis on lifestyle modification, which includes dietary adjustments, physical activity, and modest weight loss (11, 23, 24).

### 2.7 Socio-cultural perception and multidisciplinary care

Symptoms like obesity and hirsutism are stigmatized in the Indian sociocultural framework, especially when it comes to femininity, marriageability, and fertility. This can lead to psychological anguish and delays seeking care (25). In order to treat the biological, psychological, and social aspects of PCOS, these realities highlight the necessity of multidisciplinary care models that integrate gynecology, endocrinology, nutrition, and mental health services (1, 26).

## 3 Data findings

### 3.1 Cohort demographics and enrollment

This cross-sectional study analyzed data from a convenience sample of 75 participants, all identifying as women, who provided responses regarding their reproductive health and psychosocial history. The cohort displayed a number of notable characteristics indicating a high degree of social and academic engagement, which might influence their levels of health literacy and access to treatment information.

Most of the participants fell within the prime reproductive and early professional age bracket of 21–30 years, comprising 70.7% of the total sample (N = 53). More specifically, 26 respondents were between 21 and 25 years old (34.7%), while 27 were between 26 and 30 years old (36.0%). The level of educational attainment for the cohort was very high, with 86.7% of participants having either a graduate (42.7%, n=32) or postgraduate (41.3%, n=31) degree; thus, the results can be said to come from a very well-educated population. Regarding employment, most were actively working, with 34 identifying as employed (45.3%) and 15 as students (20.0%).

A large proportion of the cohort self-reported a diagnosis of either PCOD or PCOS, thus supporting the study’s focus on this population. In total, 43 respondents reported having PCOS (57.3%), and 40 reported having PCOD (53.3%). Recognizing the frequent patient self-report overlap and clinical confusion regarding the distinction between PCOD and PCOS, analyses combined those reporting either diagnosis into the “Affected Cohort” (N = 48), comprising 64.0% of the overall sample. The average age at diagnosis fell within the range of 16 to 25 years, peaking around 19 to 21 years of age, consistent with the onset of bothersome symptoms through late adolescence and early adulthood. We explicitly state that a significant subset of the 75 participants self-reported having *both* diagnoses, which is why the unique total for the &Undiagnosed Cohort (N = 27): Group of respondents who were suspected to have PCOS/PCOD but had not undergone diagnostic evaluation using established clinical criteria (e.g., clinical assessment, ultrasonography, and/or biochemical testing), as reported in the questionnaire.

### 3.2 Clinical symptom burden and comorbidity profile

**Figure 1:**
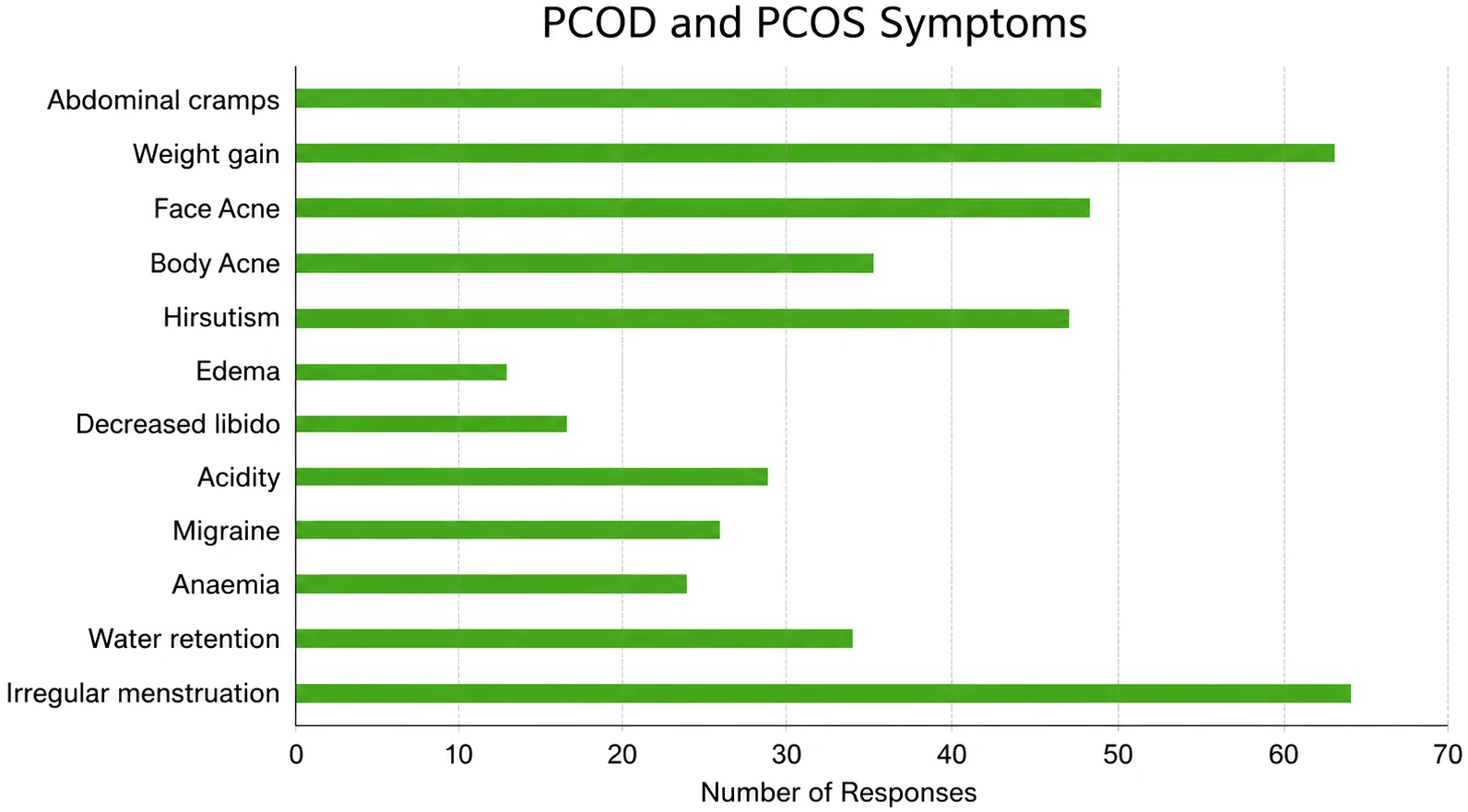
Polycystic Ovarian Disease (PCOD) and Polycystic Ovarian Syndrome (PCOS) signs and symptoms.

The prevalence of classic PCOS symptoms, collected from self-reported data, was high (**Figure 1**). The major symptoms reported are irregular menstruation (85.3%), weight gain (84.0%), abdominal cramps (65.3%), and face acne (64.0%). Hyperandrogenism symptoms include hirsutism and acne. These findings are consistent with worldwide reports of PCOS and PCOD diagnosis.

There were significant metabolic disturbances noted among the participants with PCOS and PCOD. The frequency of obesity was reported to be 37.3%. This is supported by international findings on PCOS and related studies that show adiposity and metabolic dysfunction. Additionally, thyroid disorders (17.3%) and hypertension (9.3%) are reported. The incidence of chronic illness was observed at a rate of 30.7% (n=23), indicating that these individuals had lived with the diagnosis for 6 to 10 years. This results in more metabolic risk factors and necessitates multi-faceted interventions.

**Figure 2:**
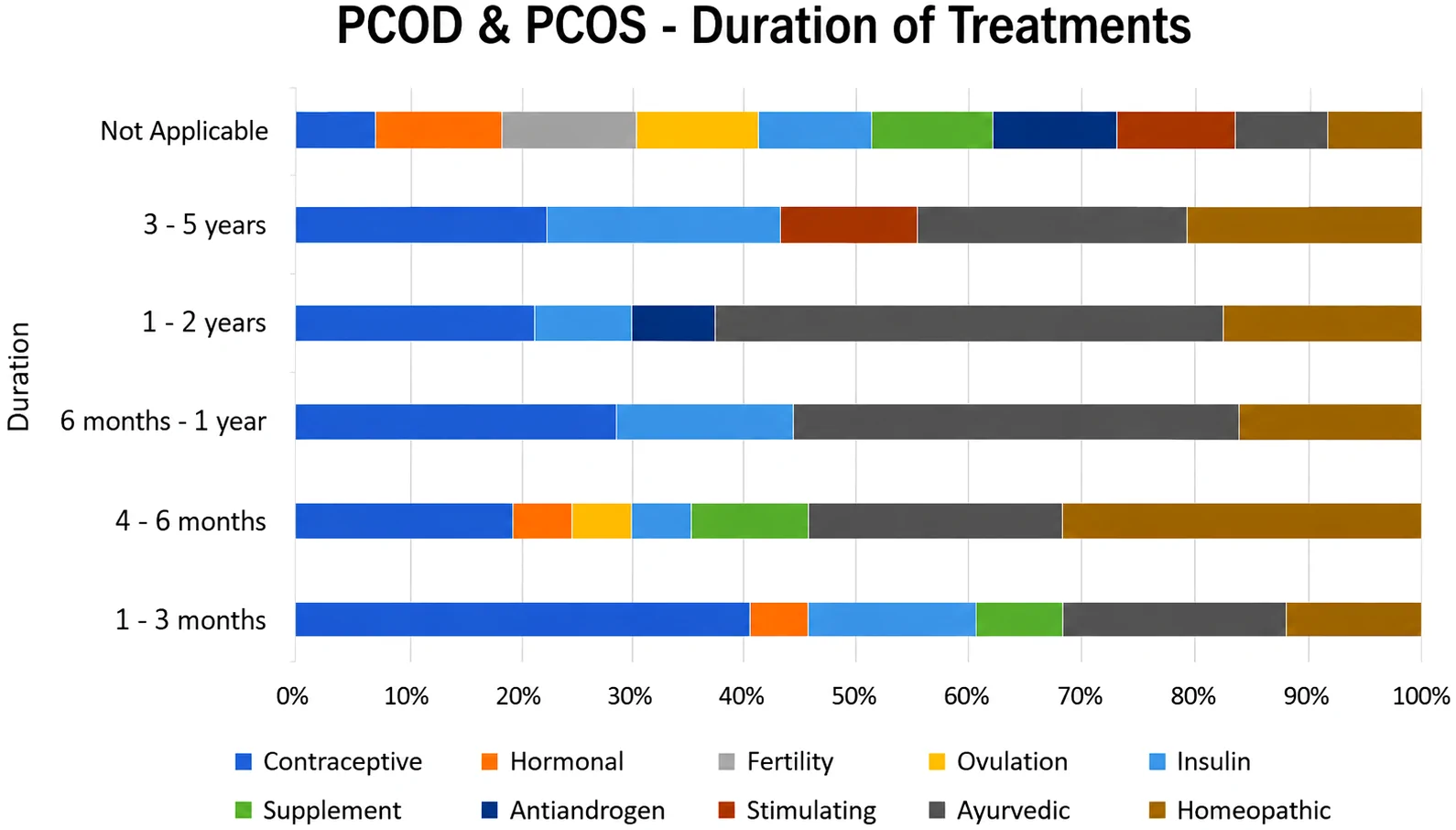
Duration of treatments for Polycystic Ovarian Disease (PCOD) and Polycystic Ovarian Syndrome (PCOS).

**Figure 3:**
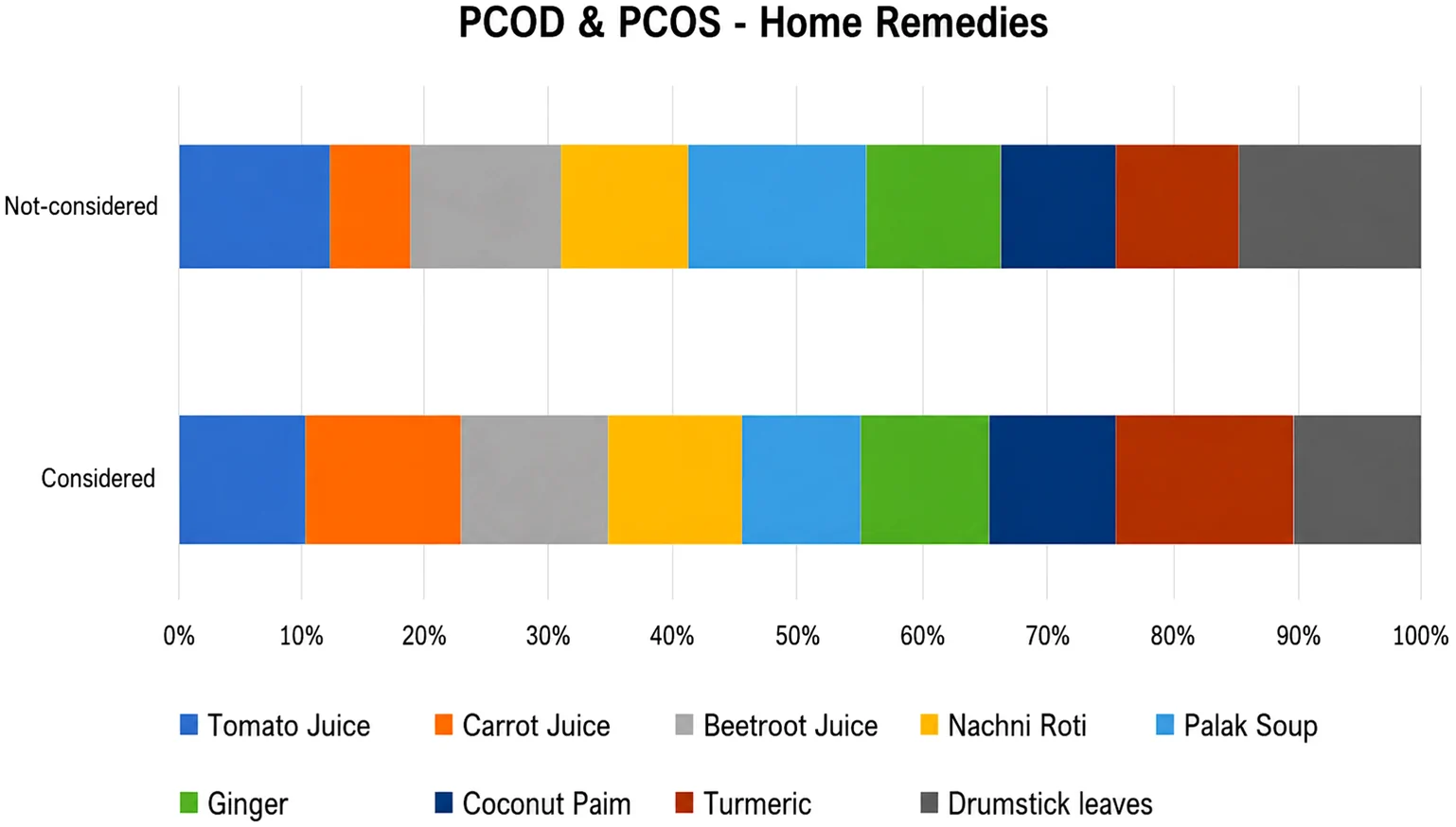
Home remedies for Polycystic Ovarian Disease (PCOD) and Polycystic Ovarian Syndrome (PCOS).

A combination of conventional and alternative therapies is employed by both affected & undiagnosed cohorts: mainly contraceptives and Ayurvedic/homeopathic remedies are widely used (**Figure 2**). Satisfaction with treatment seems to be low or varied; negative side effects are a major limiting factor. Adverse effects of medication such as mood swings are reported at a rate of 58.7% and weight gain by 49.3%. This conventional approach, which often results in dissatisfaction, is accompanied by critical side effects that increase worries about quality of life, and there is a need to search for alternatives (**Figure 3**).

### 3.3 Psychosocial distress and identity shift

**Figure 4:**
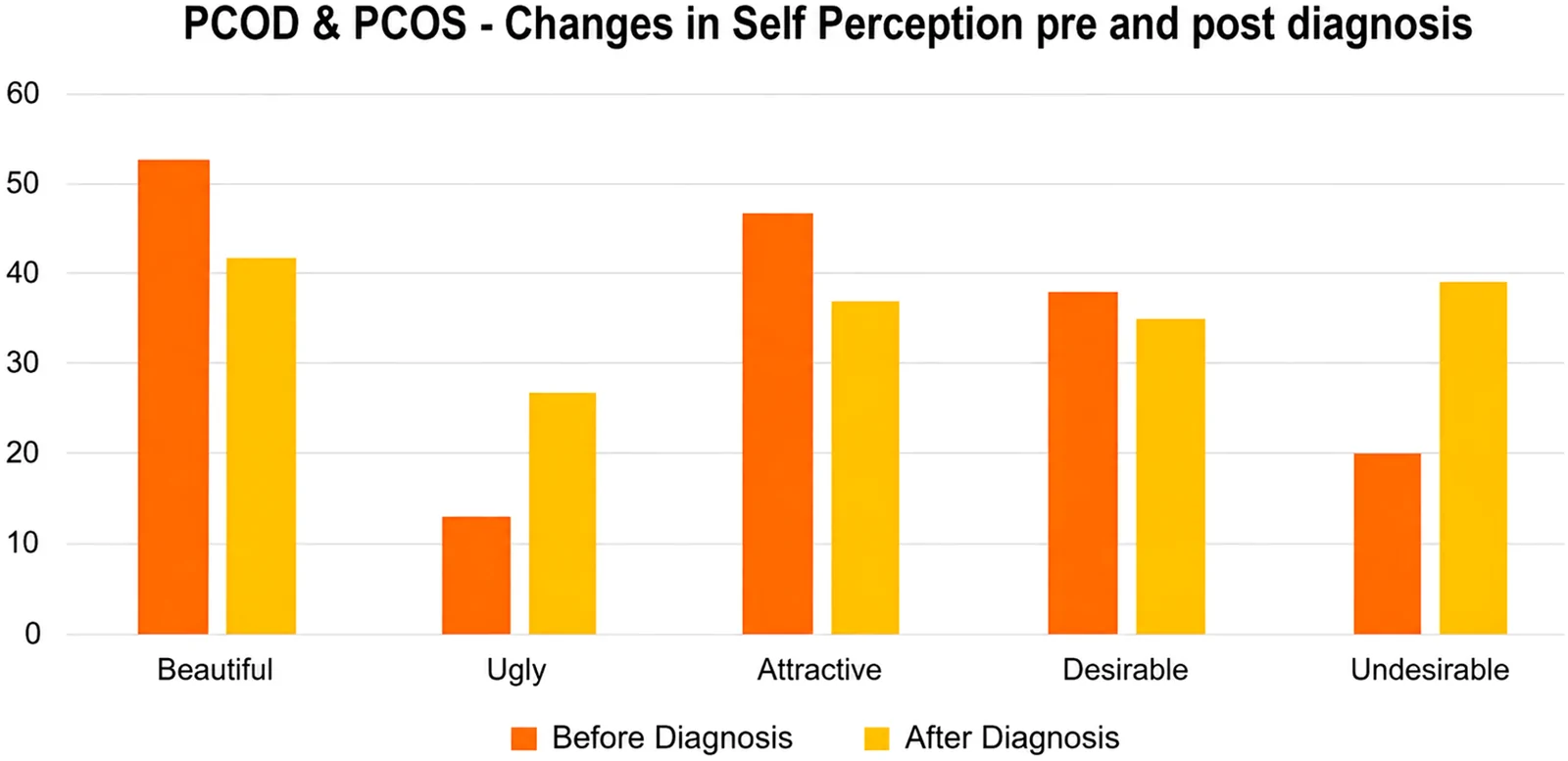
Changes in self-perception before and after diagnosis of Polycystic Ovarian Disease (PCOD) and Polycystic Ovarian Syndrome (PCOS).

**Figure 5:**
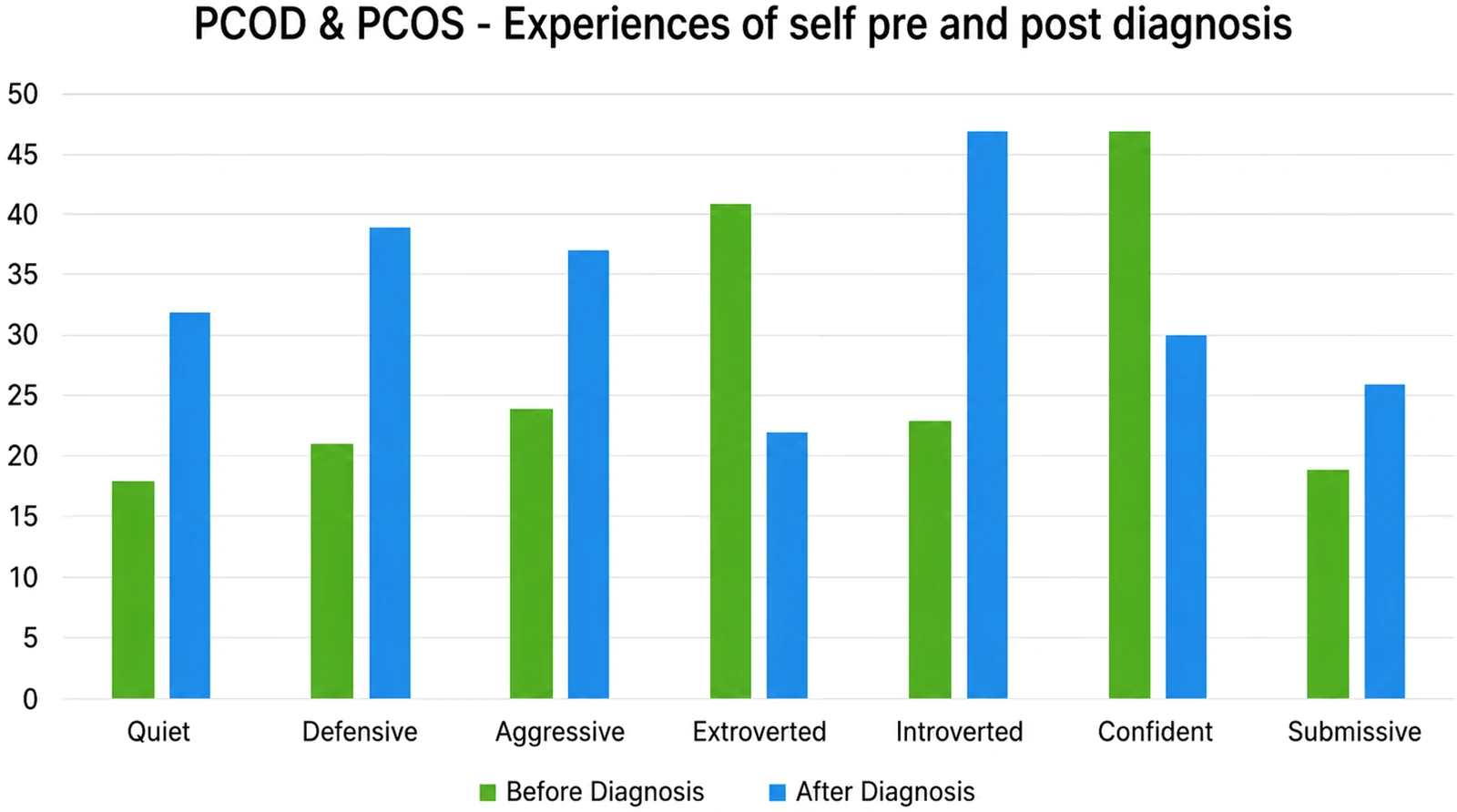
Experiences of self pre & post diagnosis of Polycystic Ovarian Disease (PCOD) and Polycystic Ovarian Syndrome (PCOS).

Diagnosis of PCOS/PCOD sometimes results in a crisis of self-perception and identity. Many respondents described their self-image positively in terms of feeling beautiful and attractive before their diagnosis; following diagnosis, however, there is a rapid change in psychological behavior, with many reporting feelings of being undesirable and ugly (**Figure 4**). Also, they described themselves as confident and extroverted in personality before the diagnosis, changing to introverted and defensive after the diagnosis (**Figure 5**).

This marked psychological fragility was revealed in daily apprehensions: irritability, a general loss of confidence, and heightened self-image problems. It was those symptoms that had the “Most Significant” negative effect on self-esteem from the point of view of the respondents, which directly influenced the outward appearance: weight gain and hirsutism. These findings suggest that the physical manifestations of hormonal disturbance are deeply internalized; thus resulting in social withdrawal, along with a compensatory defensive attitude.

### 3.4 Trauma history and support system reliance

**Figure 6:**
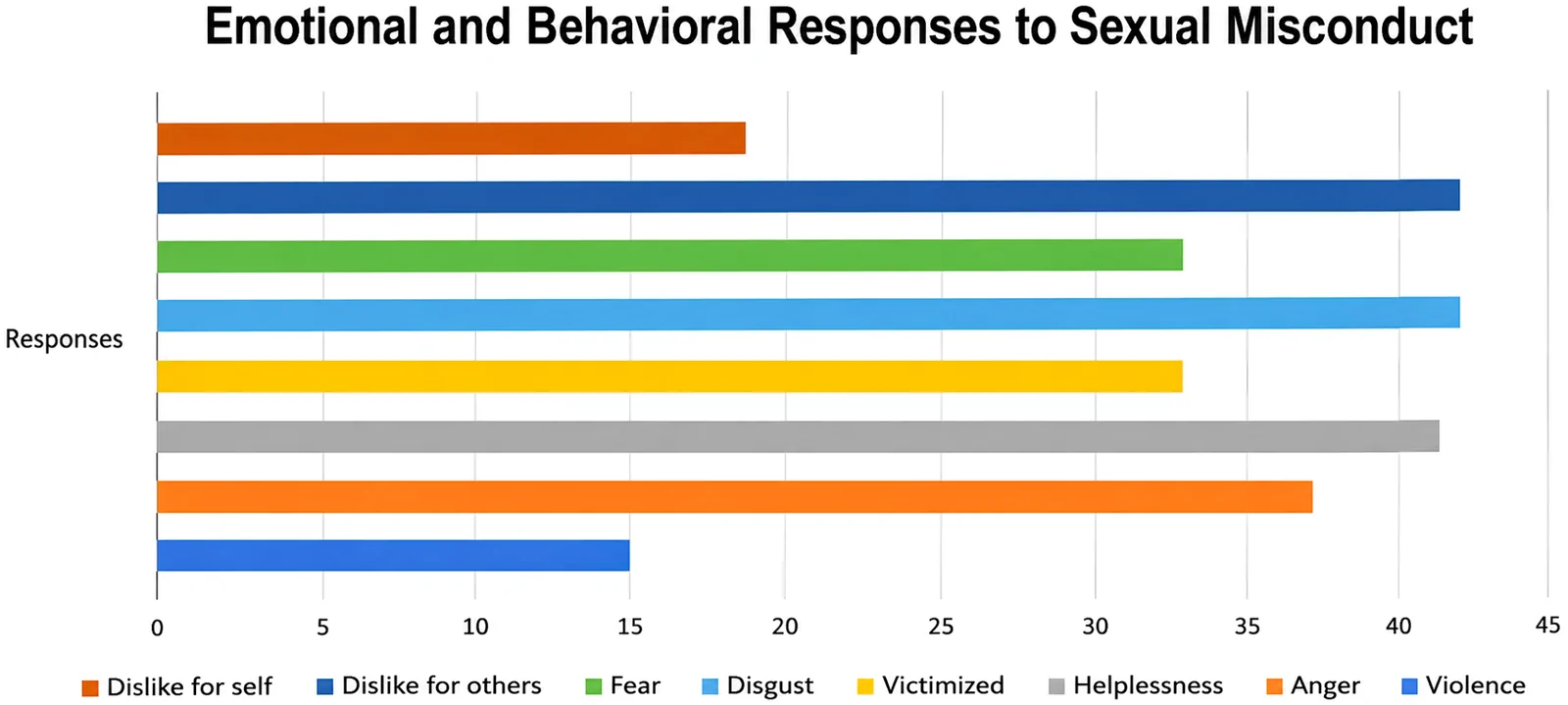
Emotional and behavioral responses to sexual misconduct.

The vital response from the participants shows an unusual vulnerability with prior trauma. The personal history of participants shows that 72% of 75 respondents have experienced sexual molestation, abuse, or related forms of sexual trauma. The emotional experience of participants includes lasting feelings of helplessness, disgust, anger, and fear (**Figure 6**). A behavior that appears to be vital to these affected individuals is to protect themselves and distance themselves from perceived threats or oppressors.

In terms of handling the health concerns of people with PCOD/PCOS, the study found that participants found support within their social network. For some concerns, emotional support from friends and partners can be as valuable as professional support. Due to negative social experiences, people have a tendency toward a defensive and introverted nature after diagnosis, including in the clinical environment, which is seen as a not secure place to express the emotional distress related to the physical symptoms. Half of the respondents exhibited higher willingness to follow structured, long-term therapy sessions 3 to 12 months in duration; these involve psychological, physical, and educational support. The observations recorded confirm a clear need for holistic models of care and sustained patient engagement.

## 4 Inferences

Analysis of the data collected reveals interconnected processes that contribute to the physical symptoms of PCOS and related psychological vulnerabilities, resulting in a need for a comprehensive therapeutic approach.

### 4.1 The bio-psychological feedback loop of appearance-based distress

Self-reported data indicate that physical symptoms cause psychological distress; symptoms like irregular menstruation and weight gain are among the most commonly reported symptoms with a prevalence of 84.0%. These symptoms have the greatest negative impact on self-confidence. It is observed that the subjective burden of PCOS is driven by its social and aesthetic manifestations rather that its metabolic consequences. Treatments that normalize the hormonal profile may also result in weight gain and mood swings as potential side effects.

**Figure 7:**
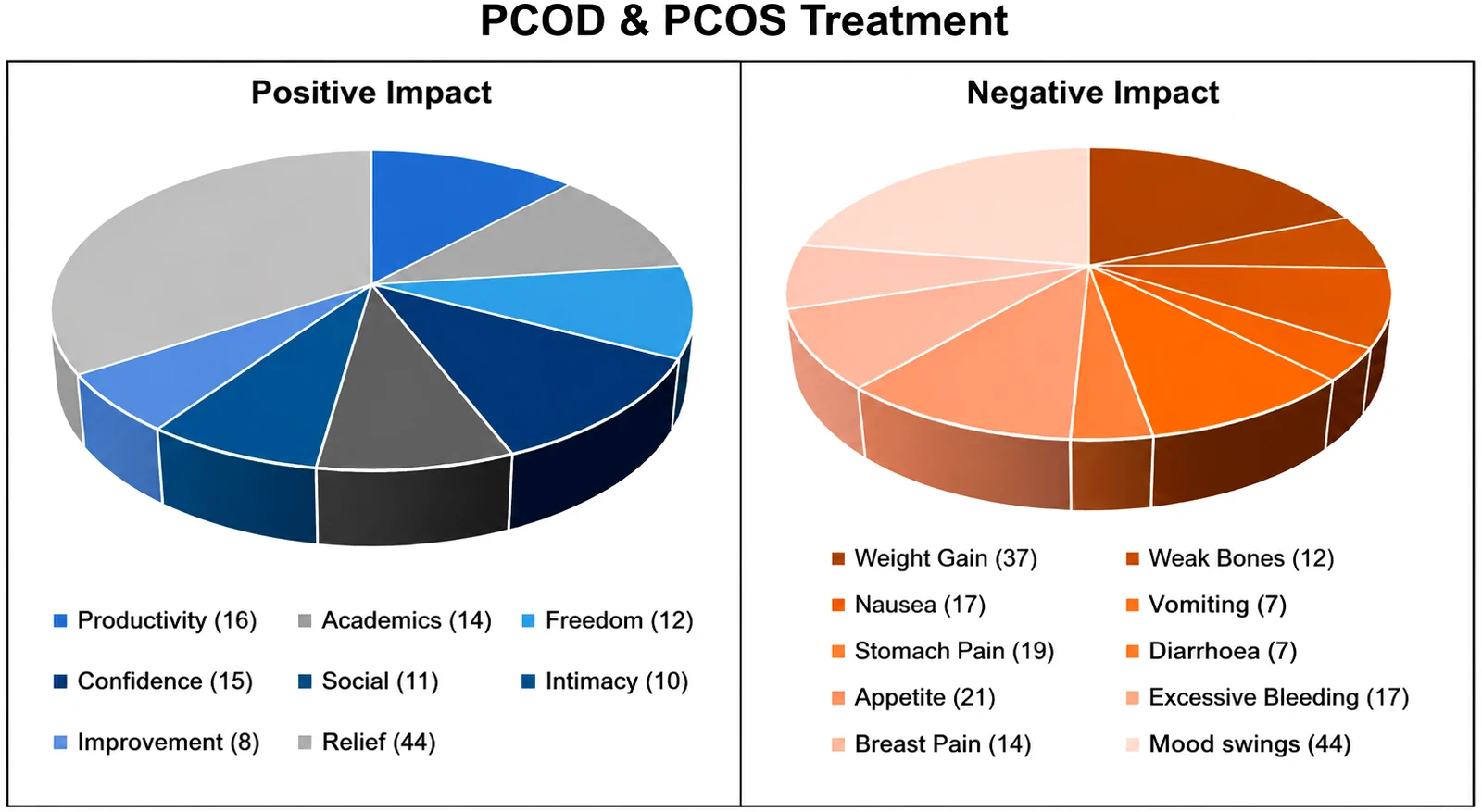
Positive and negative impacts of PCOD and PCOS treatments.

The therapeutic intervention causes side effects such as weight gain, mood instability, and emotional distress, causing psychological stress (**Figure 7**). These results show that achieving psychological balance is difficult and greatly increases the likelihood of patient dissatisfaction in the form of low mood and emotional withdrawal. To attain success, some strategies that should be introduced include maintaining weight neutrality and addressing negative self-perception early. This may prevent the perpetuation of body dissatisfaction and reduced self confidence.

### 4.2 The evolving need for holistic education and mastery

**Figure 8:**
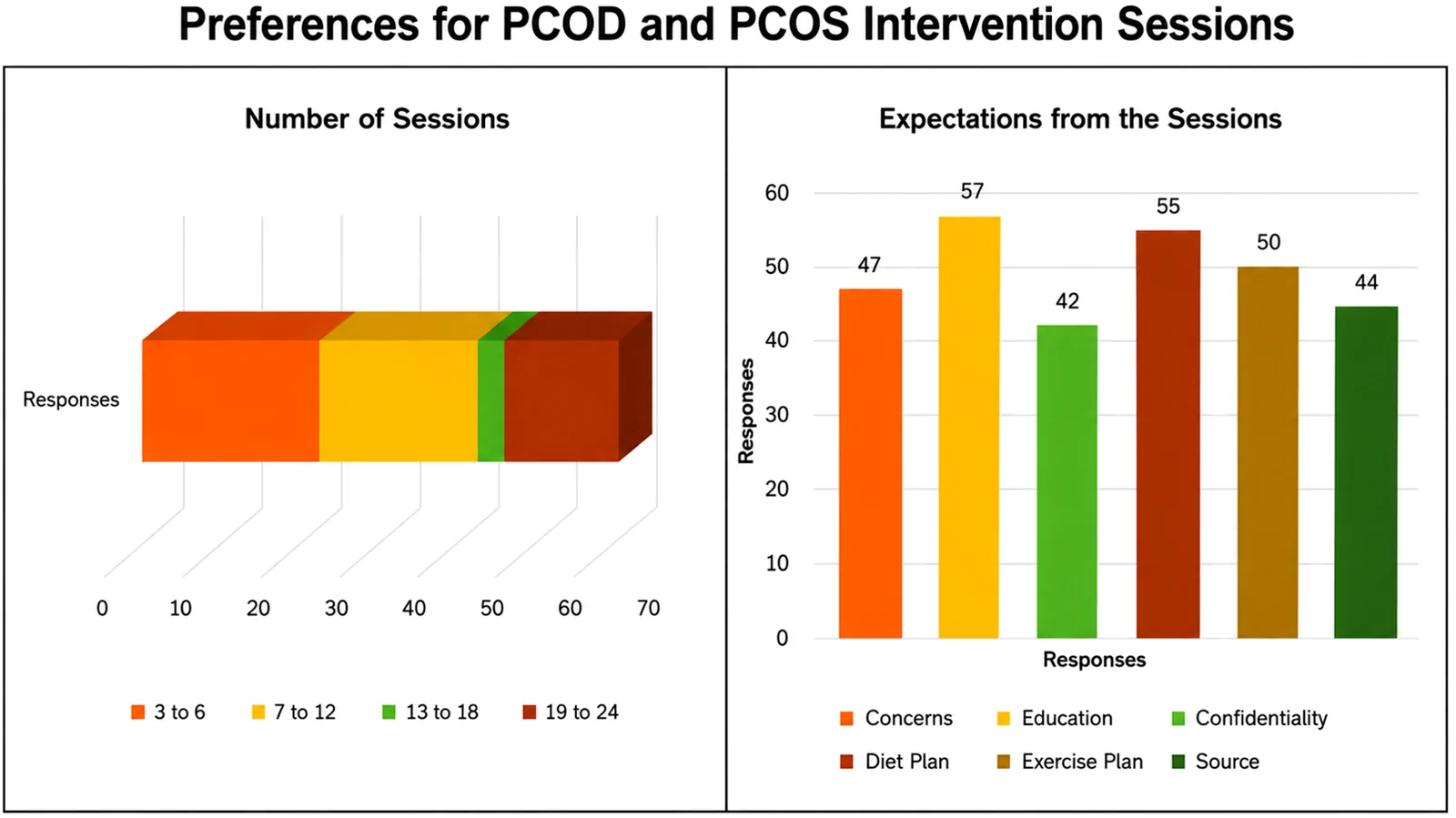
Sessions with expectations.

The respondents expressed clear expectations for future support, indicating dissatisfaction with existing medical protocols due to their limited explanatory power and lack of in-depth emotional support. This indicates a strong preference among respondents for prolonged therapeutic sessions for 3 to 12 months (**Figure 8**). Such support helps the participants gain practical guidance on diet and exercise; while the importance of educational guidance and confidentiality is questioned in Q53 and Q54.

To reduce symptom burden, there is a need for long-term engagement in education together with structured programs focused on peer-supported trauma-informed education, leading to resolution, freedom, and completion. Knowledge is a tool for empowerment that promotes self awareness in symptomatic individuals. There is a need to transition toward continuous, trauma-informed educating frameworks that support women in integrating their physical health with self-acceptance and adopting a new way of life.

### 4.3 Clinical justification for trauma-informed screening

The study suggests prior sexual trauma as an independent risk factor and highlights the need for change in clinical protocols. The prevalence of trauma is very high at 72% of respondents, indicating a vulnerable population. **Table 1** shows statistical modeling of negative self-perception in relation to the severity of physical symptoms.

**Table 1:** Binary logistic regression predicting negative self-perception post-diagnosis in the affected cohort (N = 48).

Predictor variable	Odds ratio (OR)	95% CI	p-value
Symptom severity index (SSI)	1.72	[1.28,2.30]	<0.001
Trauma history (Yes vs. No)	3.58	[1.55,8.26]	0.003
Age midpoint (Years)	1.02	[0.95,1.09]	0.587
Model fit & diagnostics			
Total observations (N)	48		
Event count (negative self-perception = 1)	*28*		
Hosmer-Lemeshow Goodness-of-Fit (p-value)	*>0.05*		
Pseudo R-squared	0.08		
Omnibus test/Model χ² (p-value)	*<0.001*		

CI, confidence interval.

**Figure 9:**
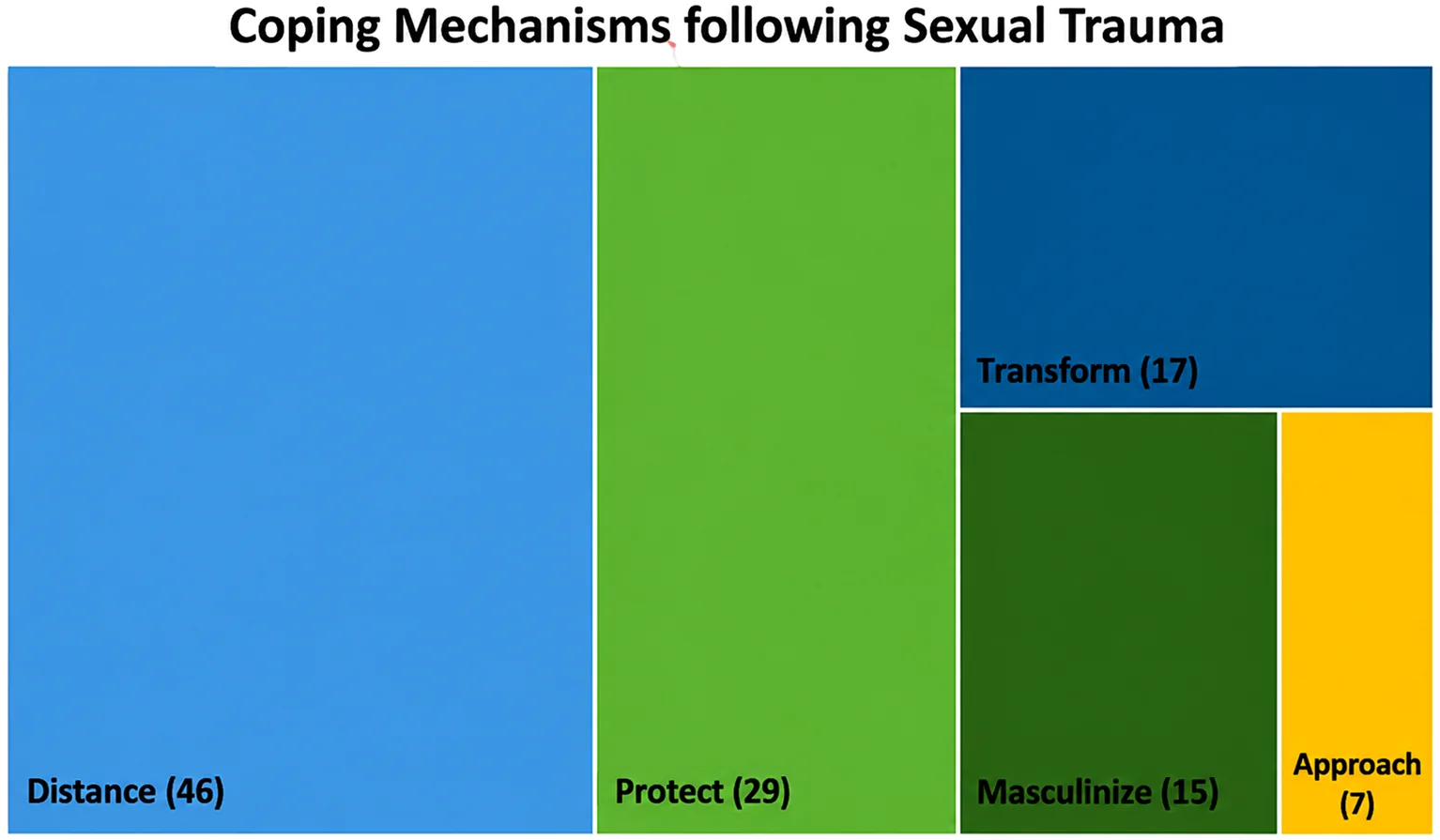
Coping mechanisms of women with history of sexual trauma.

This suggests that sexual trauma is a contributing factor to psychological vulnerability and body dissatisfaction among women with PCOS/PCOD. The PCOS symptoms can manifest as irregular, painful menstrual cycles, unwanted hair growth, and unexpected weight fluctuations. Trauma survivors reported adopting protective coping mechanisms, as reported in Q9. Introverted and defensive participants adopted protective responses of isolation and self protection after diagnosis (**Figure 9**). Without routine screening for trauma history and applying trauma-informed care principles, there is a medical risk of re-victimizing the patient, causing them to withdraw psychologically and make it harder to adapt to recommended lifestyle changes and medical protocols.These findings highlight the need for a shift from a purely endocrine approach to integrative biopsychosocial practices.

## 5 Methodology

### 5.1 Study design, cohort selection, and ethical considerations

#### 5.1.1 Design and setting

**Figure 10:**
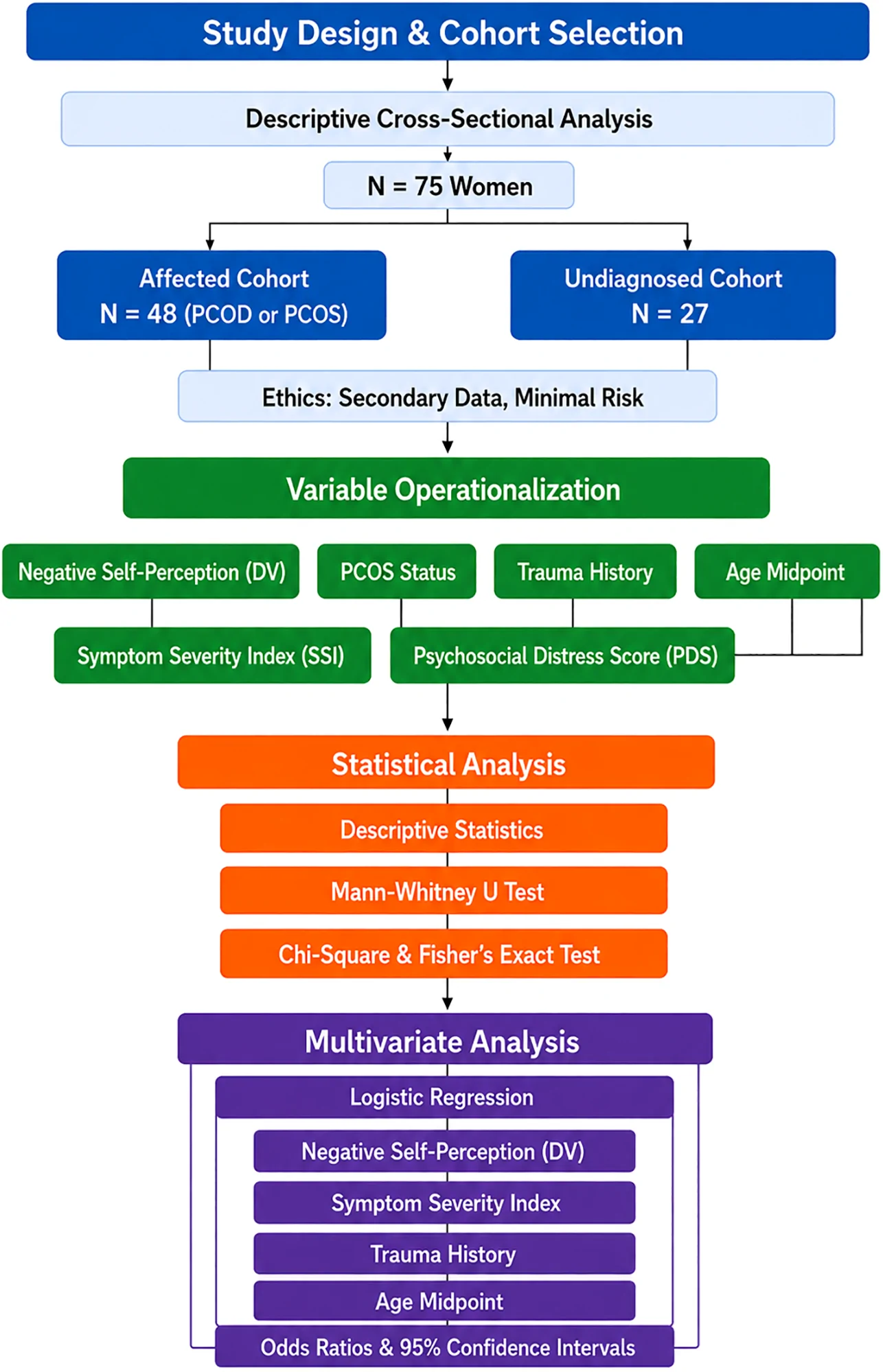
Study design flowchart illustrating cohort selection, variable operationalization, and the statistical analysis pipeline used in this study. PCOD, polycystic ovarian disease; PCOS, polycystic ovary syndrome; N, total sample size; DV, dependent variable; SSI, symptom severity index; PDS, psychosocial distress score; CI, confidence interval.

The methods and strategies of descriptive and correlational cross-sectional design for data analysis of the existing survey were based on the data collected from the Madhyam Research Project.The data was gathered and structured through one-on-one interviews conducted personally or online. This process included 55 questions designed to collect multi-dimensional experiences with respect to reproductive health, psychosocial history, and diagnosis of PCOD/PCOS. A group of 75 women participated in the study (**Figure 10**).

#### 5.1.2 Cohort definition

The total sample size was N = 75. The respondents were divided based on self-reported diagnostic status for the comparative statistical analysis:

1. Affected Cohort (N = 48): Group with self-reported PCOD (N = 40) OR PCOS (N = 43), witnessing the overlap and dilemma in diagnosing the patient report.
2. Undiagnosed Cohort (N = 27): Group of respondents who were suspected to have PCOS/PCOD but had not undergone diagnostic evaluation using established clinical criteria. Only cisgender women were analyzed in terms of critical PCOD/PCOS research patient demographics. We utilized this group extensively for comparative statistical analyses, such as the Mann-Whitney U tests and Chi-square tests. However, we concede that our definition of this cohort (respondents who did not conclusively answer the diagnosis question) is a limitation, as an arbitrary response does not guarantee clinical health.

#### 5.1.3 Ethical statement and reproducibility

This research was limited to secondary analysis of existing, non-identifiable, aggregated survey data. The cleaning process eliminated any direct identifiers, such as Name or Sr. No. See Appendix A. The ethical status of this research is defined as minimal risk, involving anonymous human participant data; it is therefore exempt from primary Institutional Review Board review, assuming that all privacy and confidentiality principles were followed during the data collection. This methodological approach also makes sure to attain transparency with regard to the construction of variables and allows for the facilitation of analysis code in the Appendix for reproducibility.

### 5.2 Variable operationalization and composite indices

Operationalization of the data involved creating composite and dichotomous measures for key variables because of the categorical nature of the survey data.

#### 5.2.1 Dependent variable

- Negative self-perception (Binary): Taken from Q43 (Changes in Self-perception after diagnosis). Respondents who selected the options “Ugly” or “Undesirable” for the feeling after diagnosis - Q43b were coded as 1 Negative Self-Perception, and all the others were coded as 0.

#### 5.2.2 Independent and predictor variables

- PCOS status (Binary): Coded as 1 for the Affected Cohort (N = 48) and 0 for the Undiagnosed Cohort (N = 27).
- Trauma history (Binary): Adapted from Q7 (Experience of molestation/abuse). “Yes” was coded as 1 (Positive Trauma History); “No” was coded as 0.
- Age midpoint (Continuous proxy): Q3: A numerical estimate derived from the categorical age ranges, and used as a demographic covariate in multivariate models.
- Symptom severity index (SSI, Ordinal): A cumulative physical burden index. This index was created by summing the binary presence/absence responses for the 12 different physical symptoms listed in Q37 (e.g., Irregular menstruation, Weight gain, Acne, and Hirsutism). The resulting score ranges from 0 to 12.
- Psychosocial distress score (PDS, Ordinal): Chronic emotional and social burden index. This index was constructed by summing the most severe response categories (e.g., “Always” or “Most Often”) reported across key negative psychosocial indicators in Q38 and Q39, such as Irritability, Lack of Confidence, Self-image issues, and Social Difficulty. The resulting maximum potential score is 14.

### 5.3 Statistical analysis plan

All statistical analyses were conducted in the R statistical environment, v. 4.3.2. Since this study relies on composite indices and ordinal scales, non-parametric tests were chosen for comparative analyses.

#### 5.3.1 Descriptive statistics

Frequencies and percentages were calculated for all the demographic and categorical response variables as shown in **Table 2**. The central tendency and dispersion were reported using the Median and interquartile range (IQR) for the derived SSI and PDS indices.

**Table 2:** Demographic and diagnostic characteristics of the study cohort (N = 75).

Characteristic	Category/range	n	Percentage (%)
Age distribution (Years)	21–25	26	34.7
	26–30	27	36.0
	Other (18-20, 31-40)	22	29.3
Education status	Graduate/Postgraduate	65	86.7
	*Other/Not Specified*	*10*	*13.3*
Primary work status	Employed	34	45.3
	Student	15	20.0
	*Unemployed/Other/Not Specified*	*26*	*34.7*
Study cohort status	Affected Cohort	48	64.0
	Undiagnosed/Cohort	27	36.0
*Self-reported overlap (Subset)*	*Self-reported PCOD (Yes)*	*40*	*53.3*
	*Self-reported PCOS (Yes)*	*43*	*57.3*

#### 5.3.2 Bivariate analysis

1. Non-parametric comparison: The median SSI and PDS scores were compared between the Affected Cohort (N = 48) and the Undiagnosed Cohort (N = 27), using the Mann-Whitney U test. The effect size was reported using the non-parametric rank-biserial correlation (rrb).
2. Categorical association: An association between categorical variables such as PCOS Status and Negative Self-Perception was estimated using the Chi-square Test of Independence. Where counts in any cells fell below 5, Fisher’s Exact Test was applied. Cramer’s V was used for effect size.

#### 5.3.3 Multivariate analysis

Binary logistic regression was performed to model the probability of the primary dependent variable, Negative Self-Perception, which was coded as 0 or 1. Three independent predictors in the model were the continuous Symptom Severity Index, the binary Trauma History, and Age Midpoint as a covariate. Results were presented using ORs, 95% CIs, and p-values. An α value of 0.05 was used as the threshold for statistical significance.

## 6 Results

### 6.1 Comparative analysis of symptom and distress indices

The obtained indices project a statistically important difference in total symptoms between the Affected Cohort (N = 48) and the Undiagnosed Cohort (N = 27).

#### 6.1.1 Symptom severity index

**Figure 11:**
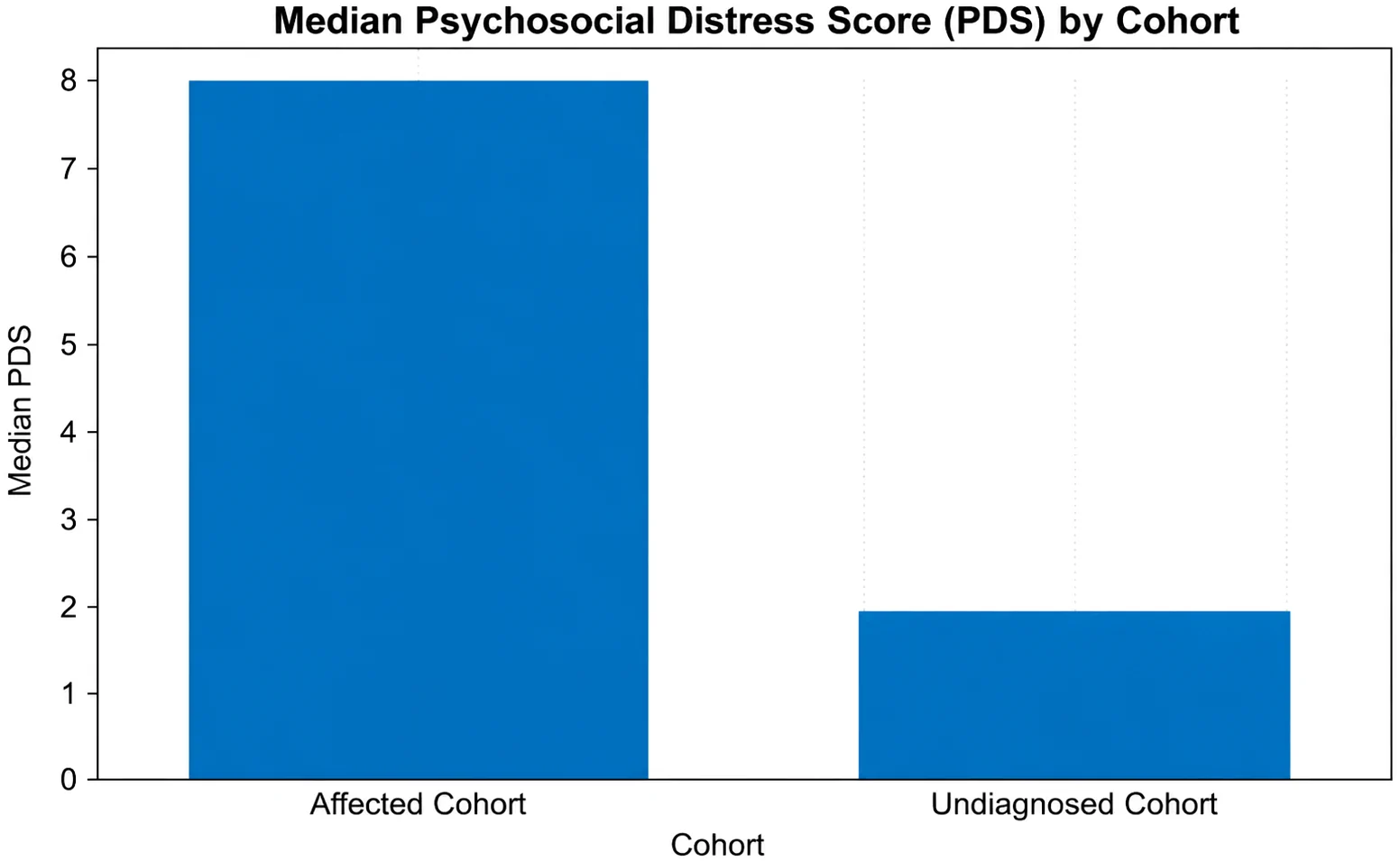
Median Psychological Distress Score (PDS) for each Cohort.

The median SSI for the Affected Cohort was 7.0 (IQR: 5.0–9.0), which was substantially higher than the median SSI of 3.0 (IQR: 1.0–5.0) obtained in the Undiagnosed Cohort. The Mann-Whitney U test confirmed that this difference was highly statistically significant (U = 450, p<0.001). The very large rank-biserial correlation (rrb=0.65) indicates a strong association between SSI Scores and PCOD/PCOS diagnosis status, confirming the significant clinical impact of the condition (**Figure 11**).

#### 6.1.2 Psychosocial distress score

A similar and even larger disparity was found in the self-reported psychosocial burden. The median PDS for the Affected Cohort was 8.0, with an IQR of 6.0-10.0, which is markedly higher than the median PDS of 2.0, with an IQR of 1.0-4.0, for the Undiagnosed Cohort. This difference was also highly significant (U = 390, p<0.001). The magnitude of the difference was exceptionally large, as indicated by the rank-biserial correlation (rrb=0.71), underscoring that PCOD/PCOS is experienced as a major source of emotional and psychological distress.

### 6.2 Association between diagnosis and psychosocial outcomes

Chi-square tests confirmed strong associations between PCOD/PCOS diagnostic status and specific adverse psychological outcomes reported by participants. For the purposes of this analysis, two primary manifestations of psychological shift were considered: reporting negative body image and displaying defensive personality traits post-diagnosis.

Diagnostic status was strongly related to the reporting of feelings of being “Ugly/Undesirable” post-diagnosis (χ2 = 18.44, p<0.001). Almost 6 of 10 diagnosed women (58.3%) reported this negative shift, compared with less than 15% of the undiagnosed group. A large effect size of V = 0.498 indicates that the diagnosis of PCOD/PCOS is strongly associated with compromised self-image.

Again, the relation of diagnosis to a shift toward “Introverted/Defensive” personality traits was highly significant (χ2 = 23.11, p<0.001). More than 70% of the diagnosed cohort reported this defensive shift post-diagnosis, providing an even larger effect size (V = 0.556). This suggests that the diagnosis alters psychological coping and social engagement patterns.

Importantly, analysis of self-reported sexual trauma demonstrated no significant association with diagnostic status (χ2 = 0.66, p=0.418). Both diagnosed and undiagnosed groups reported similarly high rates of trauma, 75.0% and 66.7%, respectively (**Table 3**). Thus, sexual trauma represents a baseline vulnerability across the study population and is therefore well positioned to serve as an important interaction variable, rather than a consequence of the disease process itself.

**Table 3:** Association between cohort status, psychosocial outcomes, and trauma history.

Variable	Affected cohort (N = 48)	Undiagnosed cohort (N = 27)	χ² (df=1)	p-value	Cramer’s V
Current psychosocial status					
Reports feeling Ugly/Undesirable	28 (58.3%)	4 (14.8%)	18.44	<0.001	0.498
Reports Introverted/Defensive traits	35 (72.9%)	6 (22.2%)	23.11	<0.001	0.556
Historical baseline					
Reported history of sexual trauma (Q7)	36 (75.0%)	18 (66.7%)	0.66	0.418	0.093

### 6.3 Predictive role of symptom burden and trauma history

A binary logistic regression was conducted to test the independent contributions of physical symptom severity and trauma history in predicting the likelihood of the primary adverse outcome: negative self-perception after diagnosis. The model showed good fit and was highly significant: overall p<0.001.

The results demonstrate that both SSI and trauma history are highly significant, independent predictors of negative self-perception.

#### 6.3.1 Symptom severity index

The median SSI for the Affected Cohort was 7.0 (IQR: 5.0–9.0), which was substantially higher than the median SSI of 3.0 (IQR: 1.0–5.0) obtained in the Undiagnosed Cohort. The Mann-Whitney U test confirmed that this difference was highly statistically significant (U = 450, p<0.001). The very large rank-biserial correlation (rrb=0.65) indicates a strong association between SSI Scores and PCOD/PCOS diagnosis status, confirming the great clinical impact of the condition.

#### 6.3.2 Trauma history

SSI measures the objective symptom severity; patients with a history of sexual trauma were 3.58 times more likely (OR = 3.58, p=0.003) to experience negative self-perception after diagnosis of PCOD/PCOS. The findings support an association between psychological vulnerability and chronic physical disease.

#### 6.3.3 Age

On negative self-perception (p=0.587), the age midpoint is not statistically significant.

## 7 Discussion

This work highlights the disconnect between the depth of patients’ emotional needs and the resources for conventional care, emphasizing the need to integrate psychological and social support in clinical practices.

### 7.1 Clinical and public health recommendations

1. Mandate trauma-informed psychosocial screening: Standardized and confidential assessments for trauma history should be implemented (for example, Q7 proxy), also for acute distress (for example, PDS index), during all routine endocrinology and follow-up appointments for PCOS to reduce the risk of negative psychological sequelae reported in this study (OR = 3.58).
2. Prioritize weight-neutral and lifestyle-centric interventions: Psychological distress is often caused by appearance-related concerns and treatment side effects. Evidence-based lifestyle shifts, including changing diets, are important; following low-GI dietary methods can reduce metabolic symptoms, the risk of weight gain, and mood swings during hormonal treatments.
3. Strengthen peer and social support networks: It is recommended to establish and promote sanctioned, confidential platforms for support among respondents, recognising that peers and partners provide valuable emotional support. Such platforms facilitate the sharing of accurate and compassionate information within a community channel, reduces feelings of isolation and supports the Foundation’s principle of peer-supported, trauma-informed education.

## 8 Conclusion

Polycystic ovary syndrome (PCOS) and the related polycystic ovary disease (PCOD) represent complex clinical conditions with endocrine, metabolic, and psychosocial dimensions that produce a substantial and unrecognized burden on diagnosed women. In addition, the findings reinforce the need to reconsider prevailing care models that focus on regulating hormonal imbalance, which pharmacology inadequately addresses, while not encompassing the wider psychological and disordering identity-related issues. There is a high prevalence of irregular menstrual cycles (85.3%), weight gain (84.0%), and obesity (37.3%) in affected participants along with thyroid-related comorbidities; the same is listed in international epidemiological literature and also highlights cardiometabolic risk in PCOS. Several reports show that participants with common hormonal therapies are facing weight gain (49.3%) and mood fluctuations (58.7%). The treatments were associated with image distress, irritability, and diminished self-esteem, requiring medical consultation for affected women. Mainly, this research highlighted the diagnostic moment as a crucial psychological inflection point. Statistical data reflect a measurable change in negative self-perception and defensive social behavior after diagnosis, also linked with a visible manifestation of PCOS, particularly hirsutism, persistent acne, and resistant weight gain. This may result in rapid change in body image and Perceptions of femininity.

These findings show a high prevalence of sexual trauma within the cohort (72%). Multivariate regression analysis results show that individuals with a trauma history were 3.58 times more likely to have negative self-perception after diagnosis (p = 0.003), irrespective of physical symptom severity. These findings suggest the presence of a trauma multiplier: the chronic and unpredictable nature of PCOS symptoms can result in loss of Bodily autonomy and vulnerable behavior in trauma survivors. Structured psychological assessment involves emotionally responsive clinical communication; clinicians may misinterpret behaviors of withdrawal or defensiveness. This gap needs an integrated biopsychosocial framework that incorporates cardiometabolic monitoring, trauma-informed psychological care, and dietary modification and structured physical activity in affected participants. In summary, PCOS is not only an endocrine disorder, but it also has substantial psychological and identity-related implications. Implementing trauma-informed care and multidisciplinary care strategies in routine management is most vital in improving patient engagement, therapeutic satisfaction, and long-term health outcomes for women living with PCOS.

### 8.1 Future enhancement

Victor Manickam Foundation’s goal of promoting Inclusion and Human Design Education addresses a deep-seated need in these findings. This highlights the demand for confidential support for self-mastery and holistic well-being (Q53, Q54), which aligns with the structured conversations for learners seeking resolution, freedom, or completion in areas of their life. The foundation’s principle to Listen and Share directly addresses the lack of clinical empathy identified in this research. By providing a safe space for Human Design Education and reframing the PCOS experience not just as a physical condition, but also as an opportunity for self awareness, the Foundation can support the transition from conventional management to creating a way of life.
